# Functional Properties of Human Stem Cell-Derived Neurons in Health and Disease

**DOI:** 10.1155/2016/4190438

**Published:** 2016-05-04

**Authors:** Jason P. Weick

**Affiliations:** Department of Neurosciences, University of New Mexico Health Science Center, Fitz Hall Room 145, 915 Camino de Salud NE, Albuquerque, NM 87131, USA

## Abstract

Stem cell-derived neurons from various source materials present unique model systems to examine the fundamental properties of central nervous system (CNS) development as well as the molecular underpinnings of disease phenotypes. In order to more accurately assess potential therapies for neurological disorders, multiple strategies have been employed in recent years to produce neuronal populations that accurately represent* in vivo* regional and transmitter phenotypes. These include new technologies such as direct conversion of somatic cell types into neurons and glia which may accelerate maturation and retain genetic hallmarks of aging. In addition, novel forms of genetic manipulations have brought human stem cells nearly on par with those of rodent with respect to gene targeting. For neurons of the CNS, the ultimate phenotypic characterization lies with their ability to recapitulate functional properties such as passive and active membrane characteristics, synaptic activity, and plasticity. These features critically depend on the coordinated expression and localization of hundreds of ion channels and receptors, as well as scaffolding and signaling molecules. In this review I will highlight the current state of knowledge regarding functional properties of human stem cell-derived neurons, with a primary focus on pluripotent stem cells. While significant advances have been made, critical hurdles must be overcome in order for this technology to support progression toward clinical applications.

## 1. Introduction

Technological innovations in cell culture models over the last decade have revolutionized the study of developmental and disease processes of the central nervous system. With the advent of induced pluripotent stem cells (iPSCs) and direct conversion techniques to create induced neurons (iNs), researchers now have the ability to examine cellular and molecular pathways in a completely human context with extraordinary genetic, pharmacological, and physiological control. For example, novel genetic engineering approaches such as zinc finger and TALE nucleases, as well as CRISPR/cas9, allow researchers to “correct” mutations in cell lines from diseased patients [1] or create targeted, disease-related mutations in “wild-type” cells [2]. Coupled with improved differentiation and specification of various neuronal and glial lineages, the analysis of* in vitro* phenotypes can be carefully tested alongside isogenic control lines in an unprecedented manner.

While much of the focus of iPSC research has been to dissect the complex molecular signaling pathways that underlie disease processes, neuroscientists must also consider the impact of genetic mutations and environmental exposure on the* functional* properties of neurons. As one of two electrically excitable cells in mammals, many disease-related phenotypes are thought to manifest as deficits in membrane excitability or synaptic communication between various neuronal populations. Many disorders such as autism, schizophrenia, and epilepsy are increasingly known as channelopathies or synaptopathies [3, 4], where proteins known to be involved with ion conductance or synaptic transmission are mutated or otherwise disrupted. The behavioral disturbances manifest in disorders of known etiology stemming from either monogenic (e.g., Rett syndrome and fragile X syndrome) or polygenic (e.g., Down syndrome) abnormalities have traditionally been thought to result from disruptions of a host of downstream effector functions, which until recently were not linked to neuronal communication. However, these pathological signaling processes are beginning to be appreciated as causing disruptions in network function by altering spike generation, integration of synaptic potentials, and/or plasticity. While* in vitro* stem cell model systems have generally lagged behind* in vivo* studies, the complex interplay between the cell-autonomous effects of deficits in neuronal function and the impact on network processing are only beginning to be elucidated even in animal models. Thus, stem cell models are now poised not only to validate* in vivo* findings but also to develop novel hypotheses regarding human-specific pathways of development and disease. However, it is imperative that stem cell biologists recognize both the opportunities and the limitations of this system to understand disease pathophysiology.

In this review I will survey recent progress regarding the functional properties demonstrated for multiple types of stem cell-derived neurons including iPSCs and iNs. I will briefly introduce the concepts regarding passive and active properties of neurons to shed light on how physiology can be used to assess neuronal maturity and identity, with a focus on glutamatergic and GABAergic neurons. Furthermore, I will discuss the ability of functional analysis to dissect pathological processes related to human disease. For my purposes, studies using hESCs and/or iPSCs have demonstrated similar findings in terms of basic functional properties [17]. Therefore, with few exceptions where applicable, I will use the more general term human pluripotent stem cell-derived neurons (hPSNs) to refer to both populations.

## 2. Functional Maturation of hPSC-Derived Neurons

### 2.1. Passive and Active Membrane Properties

The functional properties of neurons and their progenitors are driven by ion-conducting channels activated by a variety of stimuli including voltage fluctuations, secreted ligands, and mechanical forces (e.g., stretch). It is now well-established that, using differentiation techniques developed by either Zhang and colleagues [18] or Chambers and colleagues [19], a proportion of postmitotic cells display the functional hallmarks of (relatively) immature neurons compared with* in vivo* reports. The vast majority of reports using these methods suggest that, without directing specification toward particular lineages, hPSNs differentiate to forebrain progenitors which will then produce a mixed population of cortical-like glutamatergic and GABAergic neurons [20, 21], upon which we will focus our discussion. In addition, relatively pure populations of excitatory glutamatergic neurons and GABAergic interneurons can be specified using exogenous morphogens (see below) but generally express similar functional properties to “default” differentiated hPSNs.

Passive membrane properties are primarily determined by three features: (1) conductance of nongated or “leak” ion channels, (2) membrane capacitance (*C*_*m*_), and (3) conductance of the cytoplasmic milieu. At present we will ignore cytoplasmic milieu as this is not measured using standard patch clamp techniques. Commonly, ion channel conductance (*g*) is measured indirectly as the cellular input resistance (*R*_in_), which is simply the inverse of the conductance (*g* = 1/*R*_in_). Passive membrane properties are defined as those that remain* constant* during signaling processes such as action potential generation or synaptic activity. However, this is only true for a static system, most often found in “mature” neurons; developing cells display significant changes in *C*_*m*_ as neurons add new plasma membrane to elongating neuronal arbors. This has been demonstrated consistently for hPSNs, where *C*_*m*_ values significantly increase with time in culture or with addition of astrocytes (see below). *R*_in_ can generally be thought of as the number of channels per unit area of membrane and typically decreases as neurons mature, indicating the progressive addition of channels to the plasma membrane. For hPSNs, while *C*_*m*_ and *R*_in_ show robust changes over culture duration, values typically resemble neurons of late fetal stages, but not adult neurons* in vivo* and from primary cultures. For instance, with few exceptions [22], *C*_*m*_ values are twofold to fivefold lower, while *R*_in_ measurements for hPSNs are fivefold to tenfold higher than primary rodent neurons [21, 23]. This may simply reflect the relatively early time points used in most studies, as hSPNs recorded at 30 weeks* in vitro* demonstrate mean *C*_*m*_ values above 50 pF and *R*_in_ values close to mature rodent counterparts [24].

The resting membrane potential (RMP) of a cell is another proxy for determining neuronal maturity. Multiple reports have demonstrated that RMP decreases for hPSNs over prolonged periods in culture and can reach relatively mature levels after several months [7, 21, 25]. Of note regarding RMP reporting, there is wide discrepancy in the literature regarding the use of liquid junction potential (LJP) compensation to adjust RMPs based on differential ionic concentrations in the intracellular and extracellular recording solutions [26]. This can lead to wide disparities in reported RMPs, as LJP compensation can shift RMP values by 10 to 15 mV (nearly always in the negative direction) depending on solutions used. We recommend the use of LJP compensation and/or parallel recordings from primary neurons [23] to validate that RMPs recorded are accurate. RMP is largely determined by the conductance of ions through leak or nongated channels, in particular potassium (K^+^) channels in neurons. The identity of K^+^ leak channel in rodent neurons remained unknown until 1995 despite the cloning and/or characterization of most voltage- and ligand-gated channels. However, it is now known that a large family of two-pore forming KCNK channels is largely responsible for K^+^ conductance that drives RMP [27]. Because RMP of developing hPSNs decreases with time in culture, this conductance likely increases relative to *C*_*m*_. Although a functional demonstration has not been reported, gene expression studies support this notion as* KCNK3* and* KCNK10* demonstrate increasing transcript abundance over time [28].

The interplay between leak and voltage-gated potassium and sodium channels determines the intrinsic active membrane properties of neurons, including their ability to generate action potentials (APs). Nearly all studies that have quantified AP generation in hPSNs demonstrate progression from relatively immature spiking phenotypes at early time points to repetitive spike firing with more mature AP characteristics. In addition to increased number of spikes per train, AP maturation includes larger amplitude, shorter AP half width (faster spikes), and lower spiking threshold potentials [29]. However, even for the most mature hPSNs, individual APs still show prolonged half widths, moderate amplitudes, and fewer spikes/train compared with* in vivo* reports. Interestingly, the development of voltage-gated currents underlying spike generation is very similar to* in vivo* studies. For instance, voltage-gated K^+^currents are prevalent in the early developing cortex and are consistently found in progenitor cells of the ventricular and subventricular zones [30, 31]. As progenitors exit cell cycle and differentiate, only small increases are observed for total K^+^ currents, whereas the fraction of total current contributed by sustained (*I*_K_) and transient (*I*_A_) currents changes [30, 32]. *I*_A_ current is largely responsible for rapid repolarization during spike firing, allowing for repetitive AP generation. Similar to* in vivo* data, hPSNs display progressive increases in *I*_A_ current with time in culture, and this parallels their ability to generate repetitive AP trains [21]. Unlike v-gated K^+^currents, progenitor cells typically lack v-gated sodium (Na^+^) currents (*I*_Na_, [30, 31]). However, even early postmitotic neurons demonstrate relatively robust *I*_Na_ currents [8], which show modest increases with prolonged culture periods [21, 25, 33]. Together, higher *R*_in_ and RMPs, as well as lower *C*_*m*_, render hPSNs relatively more excitable than their mature counterparts. Thus, despite significantly smaller mean *I*_Na_ currents compared to adult neurons* in vivo*, hPSNs are able to fire APs in response to smaller current injections, and a substantial portion are intrinsically active. In addition, a small minority of hPSNs display properties similar to neurons* in vivo*, such as *I*_Na_ currents larger than 10 nA, LJP-adjusted RMPs near −70 mV, and *C*_*m*_ values greater than 70 pF ([34], Weick, unpublished observations). However, the variability across individual hPSNs, coverslips, differentiation methods, and laboratories leads to mean values resembling immature cells typical of late embryonic stages in rodents.

### 2.2. Glutamatergic Transmission

Rapid excitatory neurotransmission in the brain is primarily mediated by the glutamatergic* N*-methyl-D-aspartate (NMDA) and *α*-amino-3-hydroxy-5-methyl-4-isoxazolepropionic acid (AMPA) receptors. AMPARs mediate relatively fast depolarization primarily via Na^+^ influx and are composed of tetrameric assemblies of GluA1-4 subunits [35]. The GluA2 subunit is unique in that it undergoes an ADARB1-dependent posttranscriptional modification that alters the mRNA sequence encoding amino acid 607 (glutamine (Q)) to a codon encoding arginine (R) in the M2 pore forming region of the channel. Q/R editing confers several important properties to AMPARs when edited GluA2 subunits are present, including calcium (Ca^2+^) impermeability, insensitivity to block by polyamines, and reduced single channel conductance [36]. Within the hippocampus and cerebral neocortex, the vast majority of AMPA receptors contain edited GluA2, which predominantly forms heteromers with GluA1 [37, 38]. However, some reports suggest that, during early development, unedited GluA2 subunits predominate, with edited subunits being exchanged during maturation [39]. To estimate incorporation of edited GluA2 in hPSNs, Livesey et al. (2014) analyzed AMPA-evoked currents to estimate mean single channel conductance at two time points. They found significantly reduced AMPAR conductance after 5 weeks following plating compared to 2-week-old neurons. In addition, compared with five-week-old cells, neurons plated for only 2 weeks showed significantly greater sensitivity to the spermine. Together, these data suggest a developmental shift consistent with incorporation of edited GluA2 in older hPSNs [40]. Interestingly, GluA2 mRNA expression greatly exceeds that of other GluA subunits, while levels of* ADARB1* increase during hPSN differentiation [28], suggesting its expression is rate limiting for GluA2 editing consistent with previous* in vivo* studies [39, 41].

In contrast to AMPARs, NMDARs are not required for synaptic transmission in mature neurons of most brain regions but appear to play significant role in triggering the changes that underlie plasticity. This property is due largely to their ability to conduct Ca^2+^, which acts as a second messenger through activation of various downstream kinase and phosphatase cascades [42]. Similar to AMPARs, NMDARs are also thought to be comprised of tetrameric assemblies of subunits but show a strong requirement for incorporation of the NR1 (GluN1) subunit, with variable incorporation of the NR2 subunits (GluN2A-D) and/or NR3 (GluN3A-B) subunits [43]. At various excitatory synapses, including from thalamic and cortical neurons, developmental studies have shown a switch from primarily NR1/NR2B-containing to NR1/NR2A-containing receptors [44, 45]. The various subunit combinations confer critical properties to NMDARs, where NR2B-containing receptors demonstrate significantly prolonged currents and greater sensitivity to various blockers [43]. A similar developmental switch occurs for NR3 subunits, whereby NR3A predominates during embryonic and early fetal periods, and NR3B expression increases throughout adulthood [46, 47]. Using dual-SMAD inhibition in the presence of the sonic hedgehog inhibitor, Dolmetsch and colleagues demonstrated robust NMDAR currents in iPSNs [12]. Similar results were found by Gupta et al. (2013) for hESNs, which show glutamate-induced toxicity that is blocked by NMDAR antagonist MK801 [48]. Lastly, a recent study from Livesey and colleagues reported that NMDA-dependent synaptic plasticity could be induced in hPSN cultures using NMDAR activation. Fifty minutes following removal of extracellular Mg^2+^ and treatment of cultures with glycine, hPSNs were found to increase synchronicity and amplitude of spontaneous bursting, which could be blocked by AP5 [49]. These are the first data to support the idea that* in vitro*-generated hPSNs can undergo long-term changes in synaptic efficacy.

### 2.3. GABAergic Transmission

Inhibitory neurons of the CNS that express the neurotransmitter GABA come in two major flavors: (1) projection neurons and (2) interneurons (INs, not to be confused with induced neurons (iNs)). GABAergic projection neurons include the medium spiny neurons (MSNs) of the basal ganglia and Purkinje neurons of the cerebellum, which make long-range connections between distant brain regions. GABAergic INs occupy nearly all brain regions to varying degrees and make contact with excitatory neurons and other inhibitory INs locally within those areas. While INs represent only about 20% of the mammalian cortex, they are primarily responsible for maintaining excitatory-inhibitory balance in local circuits. For instance, disrupted IN function has been implicated in multiple neurological disorders including Alzheimer's, autism, epilepsy, and schizophrenia [50–53].

Early functional studies of hPSNs revealed the presence of spontaneous inhibitory synaptic activity in default differentiated cultures [21]. Gene expression [28] and immunocytochemical analyses [7] confirmed the presence of a host of genes involved with IN specification, but the precise nature of these GABAergic neurons remains to be determined. Reports using a NKX2.1-GFP reporter line have demonstrated highly enriched populations of GABAergic INs using combinations of WNT antagonism paired with treatment of progenitors with the ventralizing morphogen sonic hedgehog (SHH) [24, 54]. In contrast to default patterned cells that lack Nkx2.1 and a majority of mature IN markers [55], direct differentiation produced multiple markers of the medial ganglionic eminence (MGE), the primary origin of cortical INs [56]. In addition these cultures showed robust diversity of IN phenotypes from Nkx2.1^+^ progenitors. Postmitotic neurons expressed a range of general IN markers such as ASCL1 and DLX2 and, at later stages of development, expressed subtype-specific markers such as calretinin (CALB2), parvalbumin (PV), and somatostatin (SST). Furthermore, significant expression of functional inhibitory markers such as* GAD1* (*GAD67*),* SLC32A1* (*VGAT*), and* SLC6A1* was observed at relatively later time points. In addition, expression analysis [28] and pharmacological dissection of GABA_A_ currents themselves [57] suggest that hPSNs up to 7 weeks of age primarily express GABA_A_Rs comprised of the *α*2/3-*β*3-*γ*2 subunits, which is the predominant composition in the embryonic cortex [58]. This lies in contrast to more mature neurons that primarily express *α*1-subunit-containing GABA_A_Rs [59].

Similar to excitatory neurons, default differentiated GABAergic neurons and directed INs display progressive increases in spiking frequency and amplitude, reduced AP half width, and increased synaptic activity that is sensitive to GABA_A_R antagonists picrotoxin and bicuculline. Functional INs also display relatively immature excitable properties compared to their* in vivo* counterparts and, in some reports, appear even more delayed than their excitatory counterparts [24].* In vivo*, INs that occupy the cortex and hippocampus display the broadest range of spiking phenotypes of any neuronal population, including regular spiking (typically 10–20 Hz), fast spiking (>30 Hz), irregular spiking, delayed spiking, bursting, and stuttering [56, 60]. However, the vast majority of hPSN-derived INs appear to demonstrate regular spiking phenotypes with a small minority displaying delayed spiking properties [61]. This may be a result of deficient network activity* in vitro* where INs lack appropriate innervation from sensory fibers. One prevailing hypothesis for IN development is that while subtype specification of INs occurs transcriptionally during differentiation of MGE/CGE progenitors, maturation of functional IN properties depends on innervation from presynaptic neurons and can be highly specific to IN subtype [56, 62]. This so-called “learning on the job” may be required for hPSN-derived INs of various subtypes to achieve fully functional status.

### 2.4. Role of Glial Cells

In primary neuronal cultures as well as* in vivo*, previous studies have demonstrated that astrocytes play a significant role in promoting neuronal maturation, specifically through effects on synaptogenesis [63]. Reports using stem cell-derived neurons corroborate these findings and suggest additional roles for glia in promoting neuronal maturity. First, Johnson et al. (2007) showed that significant numbers of astrocytes differentiate from default patterned forebrain progenitors around 6-7 weeks in culture. This was coincident with the appearance of excitatory and inhibitory spontaneous postsynaptic currents (sEPSCs and sIPSCs) in hPSNs. Further, hPSNs grown on mouse cortical astrocytes developed sEPSCs/sIPSCs at significantly earlier time points compared to cultures without astrocyte addition [21]. Chen and colleagues extended these studies to show that mouse cortical astrocytes enhanced survival, arborization of neurites, AP firing, and sEPSC/sIPSC frequency and amplitude. One of the more dramatic findings from this study was the glial-induced increase in capacitance (pF); hPSNs grown for 60 days without astrocytes displayed typical *C*_*m*_ around 27 pF, whereas those in coculture achieved values around 120 pF, values more typical of primary rodent cortical and hippocampal neurons [34]. While this is likely due in part to increased dendritic complexity, it is unclear whether these results are unique to particular cell types, as other reports have shown more modest increases in hPSN capacitance in the presence of astrocytes [23].

### 2.5. *In Vitro* Network Properties

The vast majority of reports to date have focused on hPSN functions with patch clamp on a single cell level [21, 25, 33]. However, the ability of these cells to form spontaneously active networks is of great interest for conducting large-scale* in vitro* drug screening, toxicology studies, and understanding disease pathology on whole network properties. Interestingly, it was reported that networks derived from default differentiated cells rarely generate spontaneous synchronized bursts but are capable of adopting network activity when cocultured with dissociated mouse cortical neurons. It has been suggested that the lack of bursting in hPSNs may be due to the presence of a significant proportion of inhibitory interneurons [23]. This idea has been supported by reports using various secreted factors to drive glutamatergic differentiation, where more pure populations of excitatory hPSNs are capable of network bursting [49, 64]. However, it may be the case that a relatively sparse population of excitatory neurons is not capable of generating bursts or that patch clamping of individual cells is insufficient to detect relatively sparse or infrequent network activation, whereas Ca^2+^ imaging of larger numbers of cells is more sensitive ([49] but see [65]).

To identify network activation in a high throughput manner, multiple laboratories have employed multielectrode array (MEA) recording platforms. Initial studies of murine ESC-derived neurons showed that the cells were capable of forming spontaneously active networks [66, 67]. Similar to studies using whole-cell patch clamping, activity was observed to progressively develop from single spikes into more complex trains, followed by bursting [66, 67]. Validation of human ES cell-derived neuronal network formation via MEA recordings followed shortly thereafter [68]. Similar to murine systems, early forms of activity take the form of single spikes detected on various nodes that are randomly distributed; these spikes reflect axonal and/or dendritic signaling in the developing network. As the network matures, train-like spiking can give way to synchronous bursting, which is considered as mature signaling activity of the network [68, 69]. Network activity of hPSNs and mESC-derived neurons appears to be driven by excitatory and inhibitory synaptic activity as these cultures respond to AMPA/kainate, NMDA, and GABAA receptor blockers [67, 68]. Interestingly, bursting was observed on a minority of recording electrodes within the MEA platform, and those nodes that recorded bursting were clustered but also widely distributed. Thus, it may be the case that local networks within a culture are able to form bursting networks while others remain nonfunctional or simply display unique network properties. In any case, MEA recording provides a powerful tool to dissect the effects of cell type specificity, genetics, environmental exposure, and differentiation methodologies on the functional development of network behavior.

### 2.6. Functional Properties of Induced Neurons (iNs)

Despite the substantial benefits of iPSC systems, they do have significant limitations including inefficiency (typically fewer than 1% of cells are reprogrammed) and time intensity (reprogramming and differentiation typically take 3-4 months). In addition, pluripotency is associated with genetic instability and tumorigenesis [70]. To overcome these issues, direct conversion of somatic cells has been used to generate functional neurons from wild-type and diseased tissue. First developed* in vitro* using fibroblasts [71], multiple reports have also demonstrated* in vivo* conversion of astroglial cells [72, 73], which may be useful as an alternative to cell replacement therapies for regenerative purposes. The functional properties of multiple reports of human iNs (hiNs) have been summarized nicely by Chinchalongporn and colleagues [29], who suggest that most* in vitro* studies using a wide variety of combinatorial reprogramming factors report maturation levels similar to hPSNs, especially for adult somatic cell reprogramming, based on a host of passive and active properties. For instance, despite the fact that some hiNs can fire action potentials as early as 8 days after conversion, most hiNs display RMPs at relatively depolarized potentials (greater than −40 mV), low *C*_*m*_ (<40 pF), and high *R*_in_ values (1-2 MΩ). In addition, most reports use cocultures of iNs with primary neurons or astrocytes to induce synapse formation; a convincing demonstration of synapse formation using conversion of adult cells without cocultures remains elusive. In contrast, iNs converted from embryonic stem cells using single transcription factors show functional properties more similar to* in vivo* counterparts with rapid development of spiking and synaptic activity. Multiple reports have demonstrated the utility of iNs derived from stem cells to model diseases such as those associated with neuroligin-3 [74] and neurexin-1 mutations (see below, [2]) that may underlie various neurological disorders such as autism.

## 3. Functional Deficits in Neurological Disease

Multiple neurological disorders are thought to arise due to alterations in functional properties including developmental disorders such as autism spectrum disorders (ASD), Down syndrome, Dravet syndrome [8–10], Rett syndrome, schizophrenia (SCZ), and neurodegenerative disorders such as Alzheimer's (AD), amyotrophic lateral sclerosis (ALS), Huntington's disease (HD), and spinal muscular atrophy (SMA).  Table 1 summarizes many recent examinations of functional phenotypes found in diseased hPSNs. A unifying theme has begun to develop for many of these disorders, focused on the concept of excitation-inhibition balance as an endpoint to circuit-level dysfunction. While the etiology of particular disorders may lie with individual gene deficits (e.g., mutation of the MECP2 gene in Rett syndrome), the ultimate expression of dysfunction lies at the level of neuronal excitability and synaptic integration. Recent studies of human stem cell-derived neurons have identified multiple functional deficits that validate other preclinical models and, in some cases, appear highly specific.

One of the earliest examinations using hPSNs to model functional deficits was performed by Paşca et al. (2011) to study the effect of a missense mutation of the voltage-gated Ca^2+^ channel CACNA1C (Ca_v_1.2), which causes Timothy syndrome [15]. While the most severe phenotype associated with Timothy syndrome is cardiac arrhythmia, patients also suffer from developmental delay [75]. Neurons carrying Ca_v_1.2 mutations displayed a significantly prolonged AP duration as well as greater elevations in sustained intracellular [Ca^2+^]_i_. As Ca^2+^ acts as a second messenger to trigger long-term changes in cellular function, Timothy syndrome neurons displayed significant alterations in depolarization-induced gene expression compared with controls. These changes were correlated with differences in neuronal differentiation both* in vitro* and compared to transgenic mice carrying Ca_v_1.2 mutation.

Using MEA recordings, Woolf and colleagues examined the spontaneous firing properties of motor neurons derived from patients with ALS carrying SOD1^A4V^ mutation compared with unrelated wild-type cells. ALS hPSNs showed significantly greater spiking with no changes observed for passive membrane properties, which could be corrected via genetic correction of SOD1^A4V^ mutation. Interestingly, delayed rectifier potassium currents driven by the KCNQ family of Kv7 channels were markedly reduced in ALS hPSNs, and administration of the Kv7 agonist retigabine reduced hyperexcitability and caused marked hyperpolarization (~6 mV) with EC50 of 1.5 *μ*M (Table 1). Gene expression analysis suggests that the KCNQ2 channel was likely responsible for these effects, consistent with expression in cortex [5, 28]. However, previous reports have not identified the KCNQ family as linked to ALS, which may suggest a human-specific effect using ALS hPSNs. Interestingly, hyperexcitability may be a common mechanism underlying motor neuron disorders. Liu and colleagues differentiated motor neurons derived from hPSNs that carry mutations/deletions of the survival of motor neuron (SMN) genes, which lead to spinal muscular atrophy (SMA). SMA neurons displayed lower threshold of AP generation, larger spike amplitudes, and greater frequencies. In addition, SMA motor neurons showed enhanced *I*_Na_ currents with faster recovery rates, all of which were restored by expression of wild-type SMN [14]. In contrast to increased spike firing of mutant hPSNs, Kinnear and colleagues found that AP amplitude was reduced while decay was prolonged in hPSNs from Williams-Beuren syndrome iPSCs, caused by a deletion of ~25 genes on chromosome 7 [16]. Similarly, hPSNs derived from HD patients [11, 76] that display extended CAG repeats (e.g., 180) in the huntingtin gene showed reductions in spike generation that correlated with increased cell death [11]. Thus, functional phenotypes that span the spectrum from hyper- to hypoexcitability can be modeled using hPSNs to gain insight into pathological features of disease.

In addition to spiking phenotypes, multiple reports have found deficits at the synaptic level that may underlie various neurological disorders. For instance, in neurons derived from iPSCs from patients with either Rett syndrome or Phelan-McDermid syndrome (PMDS), excitatory neurotransmission was impaired as indicated by reduced amplitude and frequency of sEPSCs [12, 13]. In PMDS neurons excitatory transmission was selectively impaired, leading to a loss of E/I balance. However, E/I balance could be restored via genetic overexpression of Shank3 or treatment of cultures with IGF1, which selectively increased sEPSC amplitude and frequency [12]. In contrast, the frequency of both sEPSCs and sIPSCs was equally diminished in iPSC-derived neurons from patients with trisomy 21 (the cause of Down syndrome) [7], leading to quieter network activity overall. Importantly, physiological data was correlated with reduced immunocytochemical labeling of presynaptic synapsin-1 protein, suggesting an impairment of synaptic development regardless of transmitter phenotype. Similarly, Sudhof and colleagues used both hPSNs and induced neurons (iNs) to model psychiatric diseases (e.g., ASD and SCZ) by creating heterozygous conditional neurexin 1 (NRXN1) mutations in human embryonic stem cells (hESCs). They found that heterozygous loss-of-function NRXN1 mutations had no effect on neuronal differentiation and synaptogenesis, because labeling of Syn1 was comparable to control neurons. However, NRXN1 mutant neurons severely impaired neurotransmitter release in a stimulus-dependent manner. Interestingly, this phenotype was specific to human neurons as mouse Nrxn1a mutations exhibited no phenotype [2].

More recently, Mertens and colleagues examined the phenotypes of hPSNs differentiated from cells taken from patients with type I bipolar disorder (BD, [6]). In an elegant design this study derived cells from BD patients that showed clinical responsiveness to Li^2+^ (LR) and those that were nonresponsive (NR). Interestingly, both populations of hPSNs showed hyperexcitable properties, with increased spontaneous and evoked AP frequency and amplitude, lower threshold of activation, and increased *I*_Na_ compared to control hPSNs. Interestingly, treatment of cultures with 1 mM LiCl eliminated these differences selectively in hPSNs derived from LR patients, but not from NR patients. However, cells from NR patients did show responsiveness to the antiepileptic drug lamotrigine. The authors went on to characterize the gene expression changes induced by Li^2+^ treatment and found several potential pathways altered, including genes involved with energy homeostasis and mitochondrial function, PKA/PKC signaling, and K^+^ channels.

## 4. Conclusions and Perspectives

Since the creation of iPSCs in 2007 and iNs in 2010, they have been used to examine disease phenotypes in hundreds of publications. Thus, in a short time these platforms have had a significant impact on our understanding of disease pathology and treatment and will likely change the direction of translational research going forward. With respect to functional phenotypes, it is remarkable that fully* in vitro*-generated cells can recapitulate many aspects of disease with high degrees of specificity (Table 1). hPSNs and iNs have been shown to express a wide array of voltage-dependent and independent ion channels, the appropriate ligand-gated receptors for various neurotransmitters, and the ability to form spontaneous neural networks as well as integrate with established networks from animals separated by millions of years of evolution. In addition, reports are now emerging to suggest that signaling mechanisms in hPSNs exist to alter synaptic efficacy, a critical factor in determining fully functional neural networks. Thus, the number of conserved developmental processes that exist* in vitro* supports the furthered use of hPSNs and iNs to uncover novel and potentially human-specific molecular pathways governing functional maturation, dysfunction, and degeneration.

However, with any new tool we must caution against overinterpretation of the significance of any individual finding, particularly in light of the immature nature of the neurons derived and the variability of timing and cell type developed. As others and I have indicated [77], hPSNs and iNs achieve functional phenotypes that resemble fetal and early postnatal rodent neurons. High throughput transcriptomic studies largely agree with these findings, showing that some of the more mature hPSNs reported to date display expression profiles similar to midgestational human fetal brain tissue [78]. In addition, in many differentiation paradigms only a small percentage of neurons display synaptic markers (2–5%, [12]), and most cultures still contain a large population of progenitor cells, which do not exist in most brain regions of adults. Furthermore, iN cells typically require the use of cocultured astrocytes or primary neurons to form functional synapses [2], and direct conversion of adult cells produces neurons that are functionally less mature than those produced from neonatal cells. Interestingly, iNs from older cells appear to retain transcriptional programmes of older cells, while conversion into iPSCs typically eliminates age-related epigenetic signatures [79]. Thus, it will be critically important to improve neuronal maturation of adult-derived iNs before employing them in the study of age-related disorders. In addition, while coculturing healthy and “diseased” cells together can assist with understanding the cell-autonomous versus global network deficits, the presence of healthy cells may mask functional deficits due to paracrine and contact-mediated alterations in synaptic development.  Table 2 compares some of the main features of hPSNs and iNs to consider for experimental design.

With respect to variability in hPSN, efforts are currently underway to generate single cell transcriptomic and morphological signatures to correlate with functional properties of hPSNs in an effort to help identify subclasses of neurons. Through combined use of directed differentiation, cell sorting, and genetically encoded reporter lines, derivation of pure populations of transmitter- and functional phenotype-specific neurons is an achievable goal for some laboratories. These techniques will be particularly useful for cell-based therapies. Together with continued improvements in reducing tumorgenicity and aberrant differentiation through the use of insertion-free approaches and screening, iPSCs appear poised to revolutionize replacement therapies via functional integration with appropriate neural circuits. And, despite the cautionary notes, it is exciting to see that many stem cell researchers recognize the importance of functional assays as a complement to biochemical studies. With the improvements noted here and elsewhere [29, 80], developmental and disease modeling with human stem cells have the potential to break new ground for patient-specific therapies as well as uncover unifying mechanistic insights into seemingly disparate disease pathologies.

## Figures and Tables

**Table 1:** Dysfunction and treatment of diseased human stem cell-derived neurons.

Disease	Cell type(s)	Observed phenotypes	Refs	Treatment
Amyotrophic lateral sclerosis (ALS)	iPSC-derived motor neurons	Motor neurons derived from ALS iPSCs displayed hyperexcitability	[5]	Kv7 channel-activator retigabine reversed MN hyperexcitability

Bipolar disorder	Forebrain hPSNs	Increased AP frequency and amplitude in lithium-responsive and -nonresponsive hPSNs selectively responded to treatments (column 5)	[6]	Li^2+^ reduced hyperexcitability in hPSNs from Li^2+^-responsive patients Lamotrigine reduced hyperexcitability in Li^2+^-nonresponsive hPSNs

Down syndrome	Forebrain hPSNs	Decreased frequency (not amplitude) of spontaneous excitatory and inhibitory synaptic events	[7]	None reported

Dravet syndrome	Forebrain hPSNs	(i) Spike generation impaired in GABAergic neurons	[8]	Phenytoin reduced hyperexcitability
		(ii) Increased sodium currents	[9]	
		(iii) Hyperexcitability/spontaneous bursting resembling epileptiform activity	[10]	

Huntington's disease	Forebrain and striatal hPSNs	CAG repeat length-dependent reductions in spiking associated with increased cell death	[11]	None reported

Phelan-McDermid syndrome (22q13 deletion)	Forebrain hPSNs	Selective reduction in amplitude and frequency of spontaneous excitatory postsynaptic currents (excitation-inhibition ratio altered)	[12]	Genetic expression of Shank3 or IGF1 treatment restored EPSCs

Psychiatric disease (ASD/SCZ) (NRXN1 mutants)	Forebrain hPSNs and iNs	Impaired neurotransmitter release; reduced sEPSC frequency upregulation of presynaptic CASK protein	[2]	None reported

Rett syndrome	Glutamatergic hPSNs	Decreased activity-dependent calcium oscillations Reduced frequency and amplitude of spontaneous synaptic currents	[13]	None reported

Spinal muscular atrophy (SMA)	iPSC-derived motor neurons	Hyperexcitability and impaired neurotransmission Greater *R*_in_ and lower voltage threshold for spike induction	[14]	Genetic correction reversed phenotypes

Timothy syndrome	Forebrain hPSNs	Increased action potential width Greater elevations of intracellular calcium	[15]	None reported

Williams-Beuren syndrome	Forebrain hPSNs	Reduced AP amplitude and prolonged decay; no effect on other passive/active conductance nor mEPSCs	[16]	None reported

**Table 2:** 

Cell type	hESCs (primed)	iPSCs (primed)	iNs
Efficiency	>90%	Variable (up to 90%)	Low (2–11%)

Time to functional maturity	5 weeks +	5 weeks +	2 weeks +

Epigenome status	Embryonic (open)	Some adult modifications retained	Adult modifications maintained

Cell types produced	Neurons (many subtypes), astrocytes, oligodendrocytes	Neurons (many subtypes), astrocytes, oligodendrocytes	Primarily glutamatergic neurons

Purity of phenotypes	Heterogeneous (<80% pure)	Heterogeneous (<80% pure)	Relatively pure (>80%)

Effect of astrocytes	Accelerates maturation	Accelerates maturation	Required for functional maturation

Genetic intervention	N/A	Required	Required

Developmental studies	Appropriate	Appropriate	Less appropriate

Culture duration	Months	Months	Weeks
